# How do you wire a brain?

**DOI:** 10.3389/fnana.2013.00014

**Published:** 2013-05-28

**Authors:** Julian Budd, Zoltan Kisvarday

**Affiliations:** ^1^Department of Informatics, University of SussexBrighton, UK; ^2^Department of Anatomy, Histology and Embryology, University of DebrecenDebrecen, Hungary

Cerebral cortex is generally thought to provide the neural basis for higher cognitive and perceptual functions (see Gazzaniga et al., 2008). In cerebral cortex, billions of individual neurons, the functional units of cortex, are interconnected via a massive yet highly organized network of axonal and dendritic wiring. This wiring enables both near and distant neurons to coordinate their responses to external stimulation. Specific patterns of cortical activity generated within this network have been found to correlate with cognitive and perceptual functions (see Wang, 2010). If cortical wiring is damaged, through disease or trauma, characteristic behavioral disorders result (e.g., Seeley et al., 2009). Understanding the organizing principles of cortical wiring, therefore, represents a central goal toward explaining human cognition and perception in health and disease. Despite more than a century of endeavor, however, the organizing principles and function of cortical connectivity are not well understood.

This Research Topic presents recent progress and challenges to existing ideas about the principles concerning how cerebral cortex is wired. The publication of this collection of articles comes at a time of great excitement in the field of cortical neuroscience resulting from recent technical advances such as the more rapid tracing of cortical wiring and the ability to more precisely manipulate cortical activity experimentally. The large amount of data these new methods will yield must be tempered by the knowledge that mapping all synaptic connections or *connectome* of an individual brain represents a distant goal (see DeFelipe, 2010). In any case, the main aim of obtaining any map of cortical connectivity is to extract its underlying principles of organization—the subject of this Research Topic.

Although there are many interwoven themes in this collection of articles, we draw attention to five questions which we think will have a major bearing on the direction of future research and discuss how articles here bear on these questions.

## What is the relationship between cortical connectivity and morphology?

Without being able to prove the existence of a synaptic connnection Cajal (1899), and later Lorente de Nó (1949) were able to infer much about brain design and its underlying purpose solely from morphological data. While a number of articles in this Research Topic examine the relationship between morphology and connectivity, we use two articles here to show how this approach continues to prove useful. At the macroscopic level Mota and Herculano-Houzel (2012), propose that cortical folding is driven by white matter connectivity. Specifically, they argue that the mechanical tension generated by the pattern of connectivity of fiber bundles traveling through white matter may account for the observed pattern of cortical surface convolutions. The authors propose the degree of tension, taken as directly proportional to the morphological characteristics of the fiber bundle (i.e., axonal length and average cross-sectional area, and the proportion of efferent neurons), determines how much the cortical surface folds inwards. This model is used to explain how surface convolutions vary with brain size and how gray matter thickness varies. At the single neuron level (Cuntz, 2012), proposes that the “dendritic density field” morphological measure could be used to infer input connectivity. The author suggests dendritic arbor morphology reflects the spatial arrangement of its potential axon inputs relative to the location of its parent cell body. The shape of pyramidal and dentate gyrus granule cell dendritic arbors are explained using this approach. The author also draws attention to the benefits of morphological models for gaining insight into neuronal computation.

## What aspects of cortical connectivity are universal?

The ability to discover common principles of cortical wiring relies on acquiring a sufficiently diverse set of observations. Comparative data may, for instance, help identify and even rank the precedence of cortical wiring principles. In the context of the auditory system (Lee et al., 2011), examine the use of branched axons (collateralization) as a general wiring principle. The authors record that branched axons are commonly used for divergent processing across species and find this occurs at different levels of cortical organization. For example, they note comparative evidence for horizontal branched axons linking matched functional domains in auditory, somatosensory, and visual cortical areas. But they also report evidence for modality-specific differences in the functional use of branched axons, i.e., between axons of acoustic, somatosensory, and visual systems. To evaluate the existence of a possible multi-scale wiring principle in cerebral cortex (Budd and Kisvárday, 2012), examine evidence at single neuron, local circuit, and axon pathway scales of organization. The principle proposes that to optimize neural communication cortical wiring represents a trade-off between conserving cellular material (wire length) and minimizing conduction delay. The authors find that while there are too little data available to evaluate this hypothetical principle at the local circuit scale, strong evidence for this trade-off exists at the single neuron scale for both dendritic and axonal arbors with weaker support at the axon pathway scale.

## How are the principles of cortical wiring observed in the adult brain implemented by developmental mechanisms?

Kalil et al. (2011) review how *in vitro* dissociated neuron cultures have been used to isolate the fundamental molecular mechanisms of axon growth and branching. The authors describe recent work demonstrating how molecular guidance cues such as netrin-1 and morphogen Wnt5a alter the morphology of a developing cortical axon via the calcium-mediated reorganization of its cytoskeleton. Relative differences in the frequency of calcium transients between an axon and a branch suggest a competitive push-pull outgrowth mechanism which may underlie selective branch growth and retraction observed during *in vivo* arbor development. The mechanisms of axon branching and outgrowth are relevant to the article of (Bui Quoc et al., 2012), who report on the effects of unilateral convergent strabismus on the development of terminal arbor morphology of cortico-cortical axons linking the primary visual areas of each cerebral hemisphere. This form of abnormal sensory experience leads to the asymmetrical development of callosal terminal arbors with the creation of fewer terminal branches of a specific order in the one hemisphere compared to the other, and hence a decreased overlap between the callosal representations. The authors suggest this asymmetry prevents a unified mapping between visual hemifields required for normal visual development and binocular function. This work underscores the link between changes in the normal organization of cortical wiring and deficits in perceptual function.

## Does a universal cortical column exist and, if so, what form does it take?

The answer to this question has considerable importance for determining the dimension of a mesoscopic scale map of the Human Connectome (Bohland et al., 2009). Barrel fields in rodent primary somatosensory cortex have emerged as the *de facto* prototype for a cortical column. These cylindrically-shaped domains, readily identified by variation in cell density, supply a set of morphological coordinates with which to examine the concept of columnar processing. Feldmeyer (2012) provides a thorough review of extrinsic thalamocortical and intrinsic excitatory pathways in rodent barrel cortex. The article describes how separate parallel streams of thalamic signals are processed by the strongly vertical and recurrent excitatory connectivity within a barrel column but also describes connections beyond the column: namely, lateral interactions with neighboring barrel domains and the efferent connections with primary motor and secondary somatosensory areas and feedback to subcortical structures. While acknowledging that the existence of barrel subdomains suggests an individual barrel may not be elementary, the author cautions us not to view connectivity as static because neurons and synaptic connections are dynamically regulated by behavioral state and synaptic plasticity. A similar point is made in (Budd and Kisvárday, 2012). Comparative morphological differences between cortical areas and species also cast doubt on the notion of a universal cortical module or minicolumn (DeFelipe et al., 2002). Ichinohe (2012) describes, for example, how, immunofluorescence labeling has been used to identify the cellular composition of a honeycomb-like minicolumnar structure found in layers 1 and 2 of the rat granular retrosplenial cortex. Tracing has shown how dendritic clusters of specific cell types are grouped or segregated in relation to overlapping cortical, subicular, and thalamic axon terminal patches. The author suggests this type of structure might facilitate rapid and efficient rewiring for learning and memory tasks. What might explain this morphological diversity? Perin et al. (2013) examine theoretically the role of arbor morphology and neuronal density on the emergence of spatially overlapping clusters of recurrently connected cortical neurons. These clusters are generated by repeatedly applying the common neighbor wiring rule until the network structure stabilizes. In this rule the probability of connection between a pair of neurons is proportional to the number of connections they have in common (Perin et al., 2011). The authors report arbor extent limits the size and number of neuronal clusters, which they propose could form innate, elemental cortical groupings. Together, these articles suggest that a more flexible notion of a cortical column rather than a single, fixed dimension might provide a more accurate definition for the mesoscopic scale.

## What is best way to organize, integrate, and visualize the increasing amount of data concerning cortical wiring?

A solution to this Neuroinformatics challenge is important for progressing the discovery of cortical wiring principles. In an ambitious study (Solari and Stoner, 2011), collated, integrated, and visualized the accumulated connectivity data obtained from many published studies of primate cerebral cortex. The results can be interactively accessed on-line (http://www.frontiersin.org/files/cognitiveconsilience/index.html) and via an iPad or iPhone “app.” From the results, the authors were able to propose how particular groups of neural pathways centered around cerebral cortex might subserve specific cognitive functions. This article illustrates the importance of a comprehensive neuroanatomical assessment of a complete brain.

Overall, we think this Research Topic demonstrates the complexity and diversity of cortical organization and the wide variety of approaches that can and have been made to understand how we think and perceive. We hope the reader will find something here to stimulate their curiosity concerning a topic of considerable importance to the individual and society.
